# Grit and working memory training outcomes for children with low working memory

**DOI:** 10.1111/apa.16835

**Published:** 2023-05-17

**Authors:** Megan Spencer‐Smith, Amber Weinman, Jon Quach, Leona Pascoe, Fiona Mensah, Melissa Wake, Gehan Roberts, Peter J. Anderson

**Affiliations:** ^1^ Turner Institute for Brain and Mental Health and School of Psychological Sciences Monash University Melbourne Victoria Australia; ^2^ Clinical Sciences Theme Murdoch Children's Research Institute Melbourne Victoria Australia; ^3^ Population Health Theme Murdoch Children's Research Institute Melbourne Victoria Australia; ^4^ Melbourne Graduate School of Education The University of Melbourne Melbourne Victoria Australia; ^5^ Department of Paediatrics The University of Melbourne Melbourne Victoria Australia; ^6^ Liggins Institute The University of Auckland Auckland New Zealand; ^7^ The Centre for Community Child Health Royal Children's Hospital Melbourne Victoria Australia

**Abbreviations**
ADHD: attention deficit/hyperactiviy disorder
CI: confidence interval
Grit‐S: short grit scale
IQ: intellectual quotient
SD: standard deviation

Cognitive training programmes typically require intensive practice on challenging activities and can improve children's core cognitive abilities such as working memory for up to 6 months.^1^, ^2^ Working memory, the ability to briefly hold information in mind and work with it, is important for cognitive, behavioural and academic functioning and is more likely to be reduced in children with neurodevelopmental disorders than typically developing peers. Thus, there is great interest in interventions that improve children's working memory, such as available working memory training programmes and understanding which children benefit most. A personality trait that might influence training outcomes is grit, the perseverance and passion for long‐term goals involving consistency of interest (refrains from abandoning tasks) and perseverance of effort (displays sustained effort during challenging tasks).^3^ In children, grit has been associated with higher academic achievement^3^ and may be particularly beneficial for success in challenging activities that require sustained effort and practice. Indeed, in a study of 6‐year‐olds (*n* = 55) teacher‐reported grit was associated with improved performance on working memory training activities and outcome measures following 8 weeks of training.^4^ However, it is not clear whether grit is more likely to improve working memory in the setting of a working memory training intervention, compared with a control, or is more generally associated with improvements in working memory across childhood.

In this exploratory study, we used data from the Memory Maestros trial baseline, 6‐, 12‐ and 24‐month assessments.^1^ The trial (ACTRN12610000486022) was approved by the Royal Children's Hospital Human Research Ethics Committee, Victorian Department of Education and Training, and Melbourne Archdiocese Catholic Schools. Briefly, we screened consenting Grade 1 students (*n* = 1723) from 44 randomly selected primary schools in Melbourne, Australia, with 452 assessed as having low working memory and enrolled in the trial. Children randomised to intervention (*n* = 226) participated in the Cogmed working memory training programme, which involved practising eight of 12 computerised working memory activities for 20–25 sessions of 45 min over 5–7 weeks in small groups out of class. Two hundred and four children (90.3%) completed >20 sessions. Control children (*n* = 226) received usual teaching. Baseline characteristics were similar across groups.

Changes in verbal and visuospatial working memory from baseline to 6‐, 12‐ and 24‐month follow‐ups were examined using Backward Digits Recall and Mister X subtests from the Automated Working Memory Assessment. Grit was examined as a unique predictor of change in working memory. Teachers rated children on the 8‐item Short Grit Scale (Grit‐S) (e.g. ‘setbacks don't discourage the student’ rated from 1 ‘very like the student’ to 5 ‘not like the student’) at 24 months for children who entered the trial in terms two and four of the school year in which children were recruited (Cronbach's alpha 0.90 for the eight items in the study sample). Using separate analysis of interaction models, we explored grit as a moderator of the effect of group (intervention and control) on change in working memory from baseline to each follow‐up. Intention to treat analysis approach and raw scores were used.

The study sample did not differ from the Memory Maestros trial cohort on participant characteristics, which were similar across intervention (*n* = 90, mean age 6.99 years [0.41 SD], 47% male) and control groups (*n* = 87, mean age 6.93 years [0.45 SD], 43% male). Grit was positively associated with changes in both verbal and visuospatial working memory from baseline to 6‐, 12‐ and 24‐month follow‐ups and contributed significantly to the variance in the change in working memory scores (4%–8%). This pattern of positive association between grit and change in working memory at each follow‐up was observed for both the intervention and control groups (Table 1), with nonsignificant interactions between grit and group contributing 1% or less to the variance in the change in working memory scores. Although this exploratory study is underpowered, the very similar treatment effects in the intervention and control groups for associations between grit and change in working memory at each follow‐up, and with overlap in their confidence intervals, suggest an interaction effect is unlikely. Results persisted after adjusting for baseline attention problems (parent‐rated Conner's 3 ADHD Index) and multiple comparisons (not shown).

**TABLE 1:** Regression models for grit predicting change in verbal and visuospatial working memory from baseline to 6, 12 and 24 months by group (intervention and control).

	Change in working memory from baseline to 6 months			Change in working memory from baseline to 12 months			Change in working memory from baseline to 24 months		
	*B* [95% CI]	*β*	*sr* ^2^	*B* [95% CI]	*β*	*sr* ^2^	*B* [95% CI]	*β*	*sr* ^2^
Verbal working memory									
Univariate analysis									
Grit score	0.14 [0.07, 0.22]***	0.26	0.06	0.17 [0.10, 0.25]***	0.28	0.08	0.14 [0.04, 0.23]**	0.20	0.04
Interaction analysis									
Grit score	0.28 [0.07, 0.49]**	0.50	0.03	0.28 [0.07, 0.50]*	0.47	0.02	0.35 [0.07, 0.62]*	0.50	0.03
Group	−1.29 [−2.22, −0.35]**	−0.17	−0.03	−0.81 [−1.78, 0.17]	−0.10	−0.01	−0.42 [−1.66, 0.82]	−0.04	<−0.01
Grit score^a^ × group	−0.09 [−0.23, 0.05]	−0.24	−0.01	−0.08 [−0.22, 0.07]	−0.19	<−0.01	−0.15 [−0.33, 0.04]	−0.32	−0.01
Visuospatial working memory									
Univariate analysis									
Grit score	0.10 [0.03, 0.16]**	0.19	0.04	0.12 [0.05, 0.20]**	0.21	0.04	0.15 [0.07, 0.23]***	0.24	0.06
Interaction analysis									
Grit score	0.15 [−0.06, 0.35]	0.29	0.01	0.11 [−0.12, 0.34]	0.19	<0.01	0.28 [0.04, 0.52]*	0.46	0.02
Group	0.70 [−0.20, 1.60]	0.10	0.01	0.95 [−0.07, 1.96]	0.12	0.01	0.79 [−0.29, 1.86]	0.10	0.01
Grit score^a^ × group	−0.04 [−0.17, 0.10]	−0.12	<0.01	<0.01 [−0.15, 0.15]	0.01	<0.01	−0.10 [−0.26, 0.06]	−0.24	−0.01

*Note*: *B*, unstandardised beta coefficient is the degree of change in the outcome variable for every one unit of change in the predictor variable. *β*, standardised beta coefficient is*. sr* ^2^ squared semipartial correlation coefficient is the unique amount of variance that the predictor variable contributes to the model. Baseline working memory score included as a covariate in the models but not reported. *p < 0.05. **p < 0.01. ***p < 0.001.

Abbreviation: CI, confidence interval.

^a^ Mean centred.

In young school children with low working memory, grit was associated with improvements in working memory over a two‐year period, suggesting grit is a personality trait that may enable children to persevere in areas of cognitive weakness. We found little evidence that grit was associated with greater benefits for children who received working memory training compared with usual teaching. Study limitations include the small sample size for interaction analysis which limits the ability to make definitive conclusions from nonsignificant results, baseline IQ not assessed, brevity of the standard assessment of working memory employed, and grit measured at the 24‐month follow‐up only, although it is assumed to be a stable trait.^5^ It is important that studies powered to conduct interaction analyses replicate our study findings. Research examining methods that promote children's grit, especially in vulnerable populations, is of interest.

## FUNDING INFORMATION

This study was supported by the National Health and Medical Research Council, Australian Research Council, Victorian Government's Operational Infrastructure Support Program.

## CONFLICT OF INTEREST STATEMENT

None.

## TRIAL REGISTRATION

This study includes data from a clinical trial, ACTRN12610000486022.

## Data Availability

Data will not be made available.
